# Effect of IL-33 on pyroptosis of macrophages in mice with sepsis via NF-κB/p38 MAPK signaling pathway

**DOI:** 10.1590/ACB360501

**Published:** 2021-06-14

**Authors:** Jingnuan Ke, Guolong Cai

**Affiliations:** 1 PhD. The 2nd Clinical Medical College – Zhejiang Chinese Medical University – Hangzhou – Zhejiang, China.

**Keywords:** Interleukin-33, Sepsis, Pyroptosis, p38 Mitogen-activated Protein Kinases, MAP Kinase Signaling System, Mice

## Abstract

**Purpose:**

To demonstrate the effect of IL-33 on the macrophage pyroptosis in mice with sepsis through the NF-kB/p38 MAPK signal pathway.

**Methods:**

In total, 24 C57BL/6 mice were divided into the sham operation group (sham) and the cecal ligation and puncture group (CLP). After CLP, 24 IL-33-/- mice were divided into the IL-33-/- group and the IL-33-/- intervention group. The latter group was intraperitoneally injected with IL-33. Mouse mortality was observed after CLP. Macrophage apoptosis in peritoneal lavage fluid was detected by flow cytometry. Serum inflammatory factor level was detected by ELISA. Apoptotic protein expression and NF-κB/p38 MAKP signaling pathway protein expression were detected by qRT-PCR and Western blot.

**Results:**

Knocking out IL-33 significantly reduced the mortality of CLP mice, as well as the mRNA expression of IL-33 and the levels of serum inflammatory factors, including IL-33, IL-1β, and IL-18. It also reduced the rate of macrophage apoptosis and the expression of the apoptotic protein caspase-1 p10; increased the expression of IκBα; and reduced the protein expression of NF-κB and p38 MAPK. These effects were reversed after exogenous injection of IL-33.

**Conclusions:**

IL-33 can increase the level of macrophage pyroptosis in mice with sepsis (by activating the NF-kB/p38MAPK signal pathway) and the mortality of these mice.

## Introduction

Sepsis, a systemic organ dysfunction syndrome caused by excessive inflammatory responses to infection in the body, will further develop into septic shock in the case of abnormal cell metabolism, and abnormal circulation, characterized by high morbidity and mortality rates^1^. There is plenty of research evidence showing that sustained and excessive inflammatory responses are important factors leading to organ dysfunction and tissue damage, and also leading to causes of death in patients with sepsis^2^,^3^. Currently, organ support therapy and intensive care therapy are often applied to increase the efficacy in patients with sepsis in clinical practice, but the mortality rate remains stubbornly high^4^. As a result, deeply studying the physiopathologic mechanism of sepsis and discovering new targets for its diagnosis and treatment have become urgent challenges for scientific researchers and medical staffs. Pyroptosis, a novel pro-inflammatory programmed death, discovered in recent years, will release inflammatory factors interleukin-1β (IL-1β) and IL-18^5^. Macrophages, a class of innate immune cells that have functions such as chemotaxis and phagocytosis, can regulate inflammation and kill microorganisms, which are involved in non-specific immune responses *in vivo* 6. A study conducted by Lou *et al*.^7^ reveals that the pyroptosis rate of mononuclear cells in the peripheral blood is significantly higher in patients with sepsis than in in the ones without it, and this rate has a close correlation with the severity of sepsis and inflammation statuses. IL-33, a chromatin-related nuclear factor, exists in the nucleus under normal circumstances, whereas it is secreted extracellularly when tissue cells are damaged or sense danger^8^. Research by Halil *et al*.^9^ shows that the concentration of IL-33 in the peripheral blood is clearly increased in patients with sepsis. Based on a study by Callejas *et al*.^10^, extracellular IL-33 is able to bind to the specific receptor homolog of sulfo-transferase 2 (ST2) on cell membranes; activate mitogen-activated protein kinase (MAPK) and nuclear factor-kappa B (NF-κB) signaling pathways; induce the release of T helper type 2 (Th2) cytokines; and participate in the Th2 immune response. At present, the regulatory effect of IL-33 on the pyroptosis of macrophages through the NF-κB/p38 MAPK signaling pathway has not been studied in the case of sepsis. In this study, therefore, the role of IL-33 in the pyroptosis of macrophages in the case of sepsis was deeply researched, and the mechanism of IL-33 affecting the pyroptosis of macrophages was explored, so as to provide new clues for the clinical treatment of sepsis.

## Methods

### Animals and grouping

In this study, the research protocols were in line with the relevant provisions of the International Laboratory Animal Protection Law, and research protocols and animal-related operations were approved by the Laboratory Animal Ethics Committee of our hospital.

Specific pathogen-free (SPF) male C57BL/6 mice aged 7 to 8 weeks old were purchased from the Experimental Animal Center of the Guangzhou University of Traditional Chinese Medicine. IL-33^-/-^ mice were bought from the Mutant Mouse Regional Resource Center (USA), which were bred with C57BL/6 mice for over eight generation and confirmed to have IL-33 knockout. All mice were adaptively fed in the SPF environment with a humidity of (45±3)% at (23±2)°C under a 12/12 h light/dark cycle for 7 days before experiments, with free access to food. A total of 24 C57BL/6 mice were divided into the sham operation group (n = 12), and the model group (n = 12). IL-33^-/-^ mice were set as the IL-33^-/-^ group (n = 12) and the IL-33^-/-^ intervention group (n = 12). Models of sepsis were constructed in the model group, the IL-33^-/-^ intervention group, and the IL-33^-/-^ group by cecal ligation and puncture. In the sham operation group, the abdominal cavity was opened only, without ligation. After operation, recombinant murine IL-33 (TransGen Biotech™, Beijing, China) was intraperitoneally injected into the mice at 0.5 mg/kg in the IL-33^-/-^ intervention group, while the same volume of normal saline was injected into the mice in the model group, the sham operation group, and the IL-33^-/-^ group.

### Modeling of sepsis in mice

The mice were deprived of food for 12 h before surgery to ensure that the intestinal tract was unobstructed. In the supine position, the mice were anesthetized with an isoflurane anesthesia machine (Shenzhen RWD Life Science). Then, the abdomen was depilated and its skin was cut open along the midline. Next, the muscular layer and peritoneum were cut to find the cecum. Thereafter, the cecum was pulled out of the abdominal cavity, and thececum content was squeezed to its distal end. After that, the ileocecum and the cecum were tightly ligated at about 1/3 using 6-0 sutures. The cecum was, then, punctured twice with an 18-gauge needle, and a little intestinal content was squeezed out. Subsequently, the cecum was placed back into the abdominal cavity, and the abdominal incision was sutured. The wound was sterilized with iodophor, and a pre-heated sodium lactate ringer’s (Kelun Pharmaceutical Co., Ltd., Guizhou, China) injection was intradermally injected on the back to resuscitate the mice. The resuscitated mice were continuously fed in cages.

### Observation of survival rate of mice with sepsis

The mortality of seven mice in each group within 48 h of modeling was recorded in details, and the survival rate of mice in each group was calculated. The remaining five mice in each group were used for subsequent experiments.

### Determination of pyroptosis level of macrophages through flow cytometry

At 6 h after modeling, the mice in each group were sacrificed via cervical dislocation, followed by peritoneal lavage strictly according to aseptic operations as follows. After the mice were fixed, the abdominal skin was separated to expose the peritoneum, and phosphate-buffered saline (PBS) was injected into the abdominal cavity with a suitable syringe. Then, the mice were shaken for full lavage, and the lavage fluid was collected. This procedure was repeated three times. In order to identify macrophages, the collected lavage fluid was centrifuged at 700 g and 4°C for 5 min, then the collected cells were resuspended in 200 μL of PBS. The cells were stained with the following fluorescence-labeled antibody against mouse proteins: CD11b (14-0112, Invitrogen). Subsequently, the material was incubated with green fluorescence-labeled goat anti-rabbit secondary antibody at 37°C for 1 h in the dark, then stained with DAPI for 10 min. Immunofluorescence images were obtained using a Leica EL6000 microscope (Leica Microsystems™). Propidium iodide (PI)-positive cells (single staining) and fluorochrome-labeled inhibitor of caspases (FLICA)-positive cells (single staining) were prepared in advance. The collected peritoneal lavage fluid was centrifuged at 400 g and 4°C for5 min. The supernate was collected; reacted with an appropriate volume of erythrocyte lysis buffer for3 min; neutralized with PBS; and mixed. Afterwards, the mixture was centrifuged at 400 g and 4°C for 5 min, the supernate was discarded, and 1 mL of PBS was added to re-suspend cells. Next, 6**´**10^5^ cells were added to flow tubes and centrifuged at 400 g and 4°C for 5 min. Thereafter, the supernate was discarded, and 290 μL of flow buffer and 10 μL of FLICA were added, mixed and let stand in a dark place at room temperature for 40 min. After that, 0.5 μL of antibody was added and mixed, followed by standing in the dark at room temperature for 20 min. Next, 2 mL of flow buffer was added and centrifuged at 400 g and 4°C for 5 min. Afterwards, the supernate was discarded, and the cells were washed twice with an appropriate volume of flow buffer, re-suspended with an appropriate volume of flow buffer, added with 0.5 μL of PI, and placed ona flow cytometry (BD Accuri C6, BD) for detecting the pyroptosis level of macrophages.

### Detection of the content of inflammatory factors via enzyme-linked immunosorbent assay (ELISA)

Before the mice were sacrificed, they were anesthetized with an isoflurane anesthesia machine. After the blood was taken from the eyeballs, it was allowed to stand at 4°C for 1 h, and then centrifuged for 10 min to separate the serum. IL-1β, IL-18, and IL-33 ELISA kits (Nanjing Jiancheng Bioengineering Research Institute, Nanjing, China) were utilized to detect the content of inflammatory factors IL-1β, IL-18, and IL-33 in the serum of mice in each group, strictly according to the instructions. The optical density (OD) value of each group of samples was read using a microplate reader at 450 nm. Standard curves were plotted, and the contents of IL-1β, IL-18, and IL-33 in each group of samples were calculated.

### Measurement of messenger ribonucleic acid (mRNA) expression levels via quantitative polymerase chain reaction (qPCR) assay

After the collected peritoneal lavage fluid was centrifuged at 400 g and 4°C for 5 min, the supernatant was taken, treated with an appropriate volume of erythrocyte lysis buffer for 3 min, neutralized with PBS and mixed, followed by centrifugation at 400 g at 4°C for 5 min. After that, the supernatant was discarded, and the cells were re-suspended with 1 mL of PBS. Total RNAs were extracted from macrophages by the TRIzol (Invitrogen) method, and their OD value was determined. Then, RNAs with good quality were selected for subsequent experiments. The aforementioned RNA samples were reversely transcribed using a reverse transcription kit (TaKaRa) under conditions of 43°C for 15 min and 94°C for 2 min. The primers were synthesized by Invitrogen. The sequences are shown in Table 1, with glyceraldehyde-3-phosphate dehydrogenase as an internal reference. The qPCR system was prepared. The reaction conditions were as follows: denaturation at 95°C for 5 min; denaturation at 95°C for 30 s; annealing at 60°C for 30 s; and extension at 72°Cfor 1 min for 30 cycles, followed by extension at 72°C for5 min for termination. The relative mRNA expression level of IL-33 was calculated using 2^-ΔΔCt^.

**Table 1:** Primer sequences.

Gene		Sequence of PCR primers
IL-33	Sense	CCTGGCTCTTGCTTGCCTT
	Antisense	GGTCTTGTGTGATGTTGCTCA
GAPDH	Sense	TATCGGACGCCTGGTTAC
	Antisense	TTCCCATTCTCAGCCTTG

### Detection of expression levels of proteins through Western blotting assay

Following the centrifugation of the collected peritoneal lavage fluid at 400 g and 4°C for 5 min, the supernate was collected; reacted with an appropriate volume of erythrocyte lysis buffer for 3 min; neutralized with PBS; mixed, and centrifuged at 400 g and 4°C for 5 min. Thereafter, the supernate was discarded, the cells were re-suspended with 1 mL of PBS, added with an appropriate volume of RIPA lysis buffer, and centrifuged at 12.000 rpm and 4°C for 10 min. After that, the supernatant, *i. e*., protein samples, was collected. The proteins were quantified, and a protein loading system with equal concentration was prepared and boiled at 95°C for 15 min in order to inactivate the proteins, which were separated by SDS-PAGE, and transferred to a PVDF membrane at 100 V for 90 min. After that, protein bands were blocked with 5% skimmed milk powder for 2 h. The target bands were cut; incubated with pro-caspase-1, pro-caspase-1 p10, NF-κB inhibitor alpha (IκB-α), NF-κB p65, phosphorylated (p)-p38 MAPK, p38 MAPK, and GAPDH (CST, diluted at 1:1000) monoclonal antibodies at 4°C overnight; washed with Tris-buffered saline-Tween 20 (TBST) three times (5 min/time); and incubated with horseradish peroxidase-conjugated secondary antibody (Beyotime) at room temperature for 1 h, followed by washing with TBST three times (5 min/time). Lastly, ECL solution was added, and a developing machine was utilized for image development and analysis of protein.

### Statistical analysis

The data in this study were expressed as median and interquartile range, and analyzed by SPSS 22.0 software (SPSS Inc.™, Chicago, IL, USA). For survival analysis, a Mentex-Cox test was adopted. The chi-square test was used for enumeration data. Analysis of variance was employed for comparison among groups. p < 0.05 suggested a statistically significant difference.

## Results

### Effect of IL-33 on the mice survival rate

**Figure 1:**
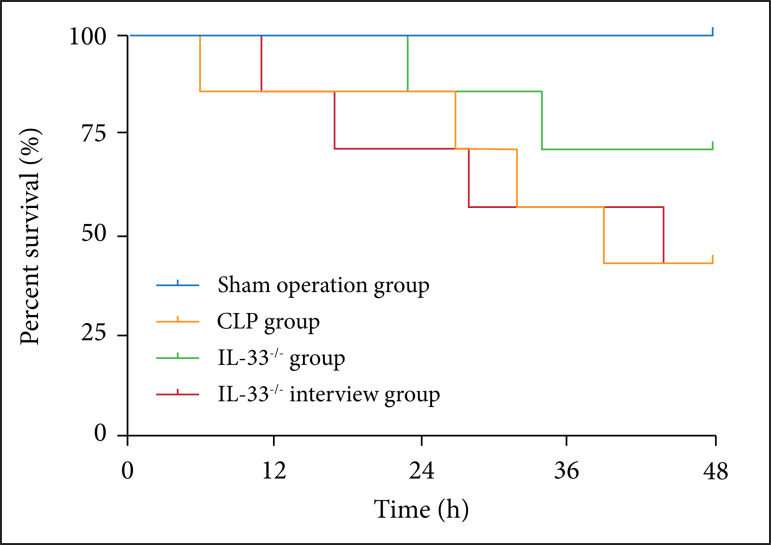
Survival rate of mice in sham operation, model, IL-33^-/-^, and IL-33^-/-^ intervention groups. Survival rate of mice in sham operation, model, IL-33^-/-^, andIL-33^-/-^ intervention groups (n = 7). Survival rate of mice in sham operation, model, IL-33^-/-^, and IL-33^-/-^ intervention groups (n = 7); ^&&^p < 0.01 *vs.* sham operation group; ^^^^p < 0.01 *vs.* model group; ^##^p < 0.01 *vs.* IL-33^-/-^ group.

The mice survival rate in each group was recorded within 48 h after modeling. It was found that this rate of the mice in the model group was obviously lower than that of the ones in the sham operation group (p < 0.01). It was also overtly lower in the IL-33^-/-^ intervention group than in the IL-33^-/-^ group (p < 0.01), while notably higher in the IL-33^-/-^ group than in the model group (p < 0.01) (Fig. 1).

### Macrophage identification and mRNA expression level of IL-33 in mice peritoneal lavage fluid

**Figure 2:**
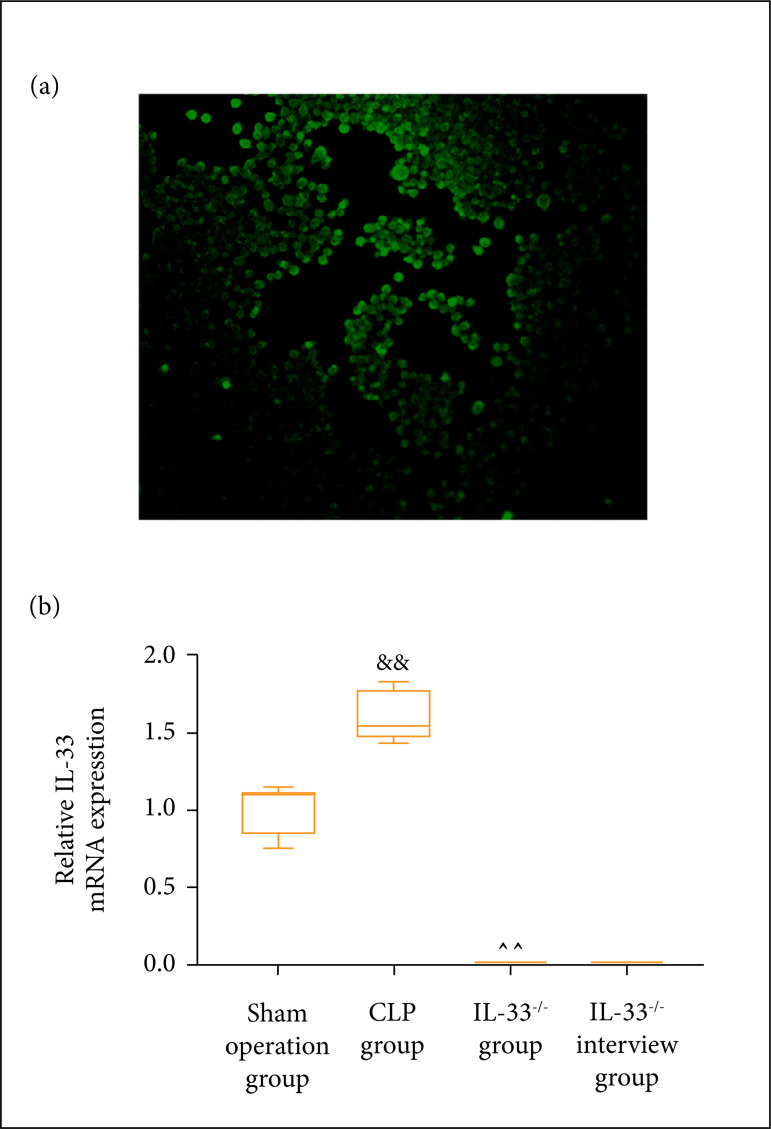
Macrophage identification and mRNA expression level of IL-33 in mice peritoneal lavage fluid. **(a)** Immunofluorescence to detect CD11b expression in peritoneal lavage fluid; **(b)** mRNA expression level of IL-33 in sham operation, model,IL-33^-/-^, and IL-33^-/-^ intervention groups (n = 5); ^&&^p < 0.01 *vs.* sham operation group; ^^^^p < 0.01 *vs.* model group;^##^p < 0.01 *vs.* IL-33^-/-^ group.

The expression of the macrophage marker CD11b in the peritoneal lavage fluid was identified by immunofluorescence. As shown in Fig. 2a, CD11b was positively expressed, indicating that the isolated cells were macrophages. The mRNA expression level of IL-33 in the macrophages in the peritoneal lavage fluid was measured through qPCR in each group of mice. The results showed (Fig. 2b) that, in comparison with the model group, both sham operation and IL-33^-/-^groups had a lowered mRNA expression level of IL-33 in macrophages (p < 0.01). IL-33 mRNA expression of the IL-33^-/-^ intervention group was significantly higher than the one of IL-33^-/-^ group (Fig. 2).

### Content of serum inflammatory factors in mice

**Figure 3:**
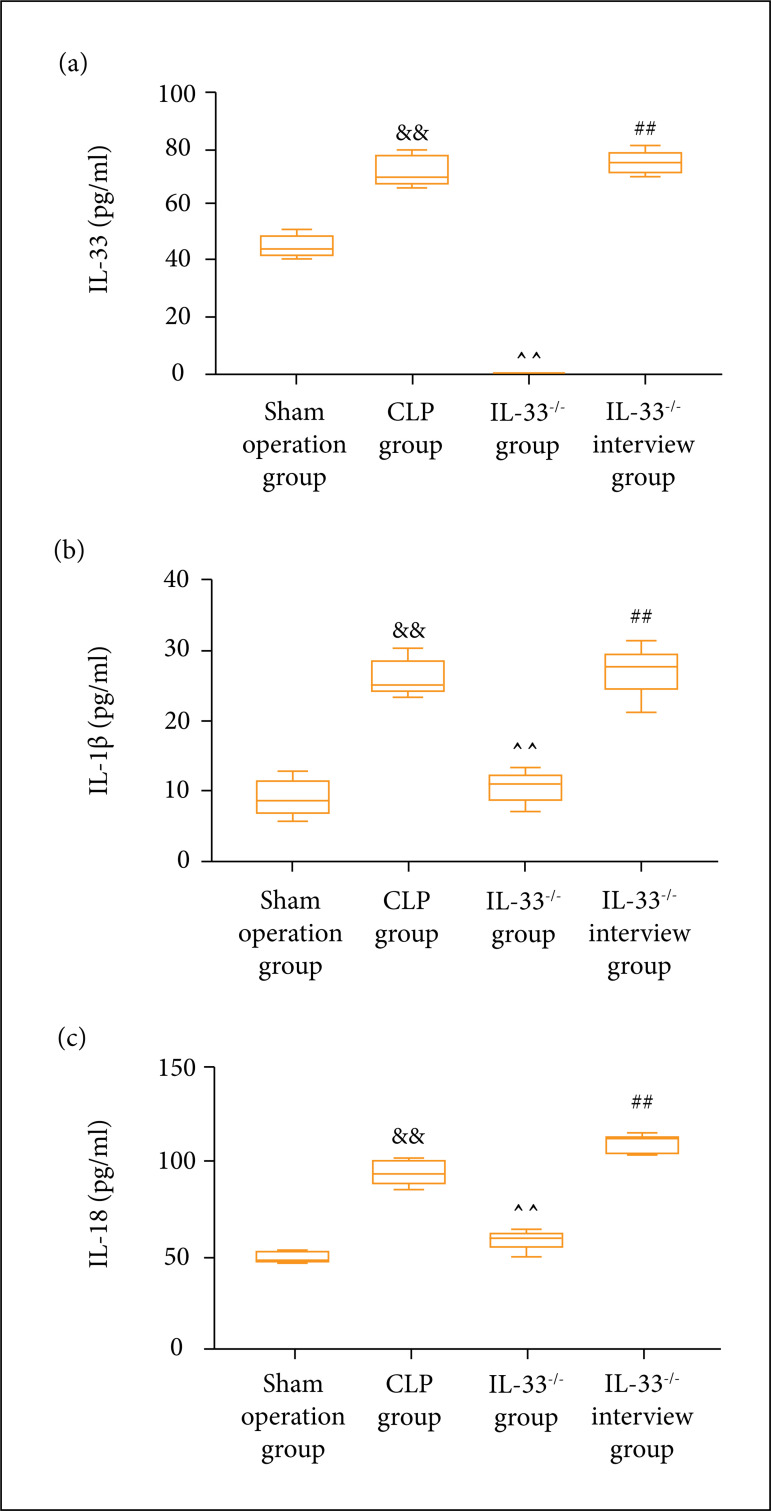
Content of inflammatory factors detected via ELISA. (a) Content of IL-33; **(b)** Content of IL-1β; **(c)** content of IL-18. Content of IL-33,IL-1β, and IL-18 in the serum of mice in sham operation, model, IL-33^-/-^, and IL-33^-/-^ intervention groups (n = 5); ^&&^p < 0.01 *vs.* sham operation group; ^^^^p < 0.01 *vs.* model group; ^##^p < 0.01 *vs.* IL-33^-/-^ group.

The ELISA kits were used to detect the content of inflammatory factors (IL-33, IL-1β, and IL-18) in the serum of mice in each group. It was discovered that the content of IL-33, IL-1β, and IL-18 was observably raised in the model group in comparison to the sham operation group (p < 0.01). It was also prominently higher in the IL-33^-/-^ invention group than in the IL-33^-/-^ group (p < 0.01), while lowered in the IL-33^-/-^ group in comparison to the model group (p < 0.01) (Fig. 3).

### Pyroptosis level of macrophages in mice peritoneal lavage fluid

**Figure 4:**
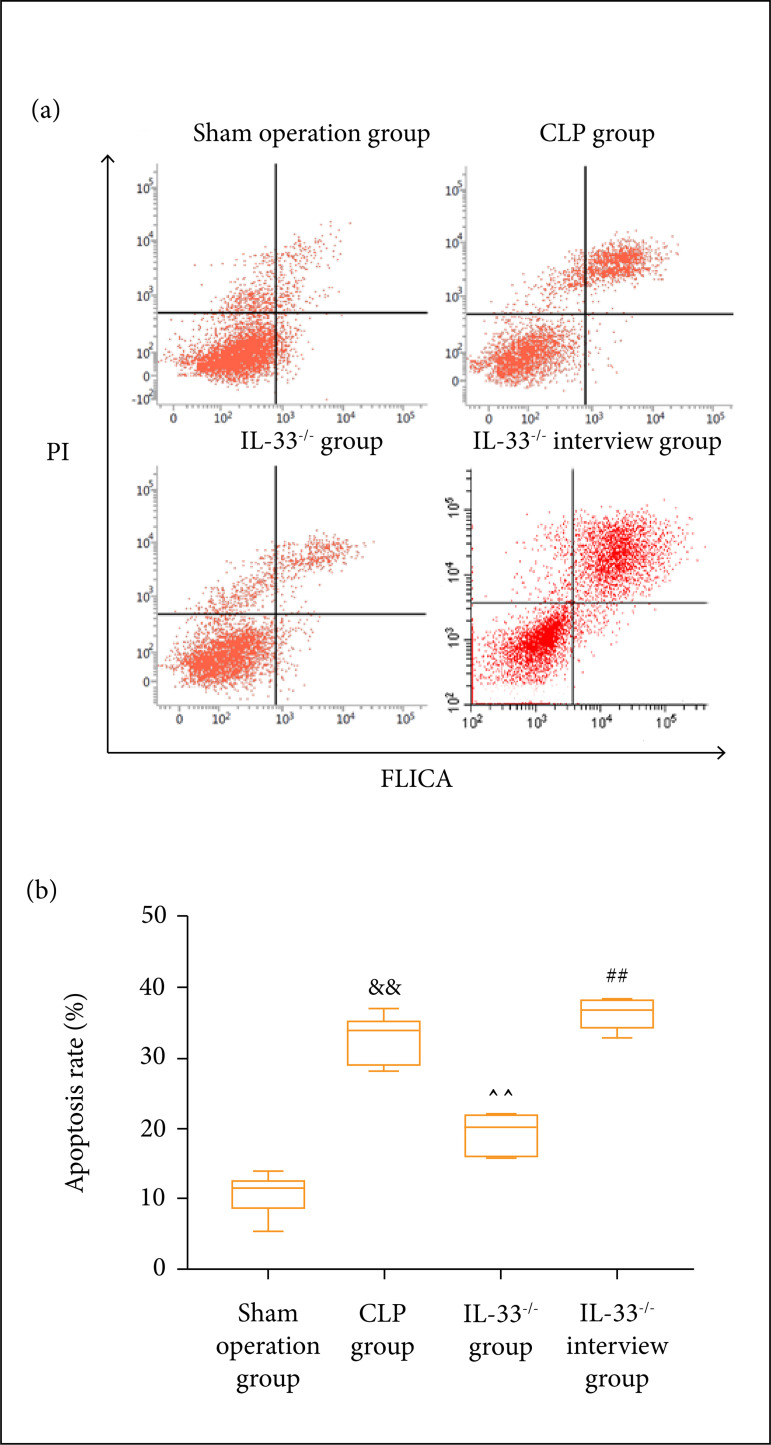
Pyroptosis level of macrophages in mice detected through flow cytometry. (a) Results of flow cytometry; (b) statistical graph. The pyroptosis level of mice macrophages in sham operation, model,IL-33^-/^, and IL-33^-/-^ intervention groups (n = 5); ^&&^p < 0.01 *vs.* sham operation group; ^^^^p < 0.01 *vs.* model group;^##^p < 0.01 *vs.* IL-33^-/-^ group.

The pyroptosis level of macrophages in the peritoneal lavage fluid was detected by flow cytometry in each group of mice. The results revealed that the pyroptosis level of macrophages rose considerably in the model group in comparison to the sham operation group (p < 0.01). Compared to the IL-33^-/-^ group, it was higher in the IL-33^-/-^ invention group, and compared to the model group, it was significantly lower in theIL-33^-/-^ group (Fig. 4).

### Expression level of pyroptosis-related proteins in mice peritoneal lavage fluid

**Figure 5:**
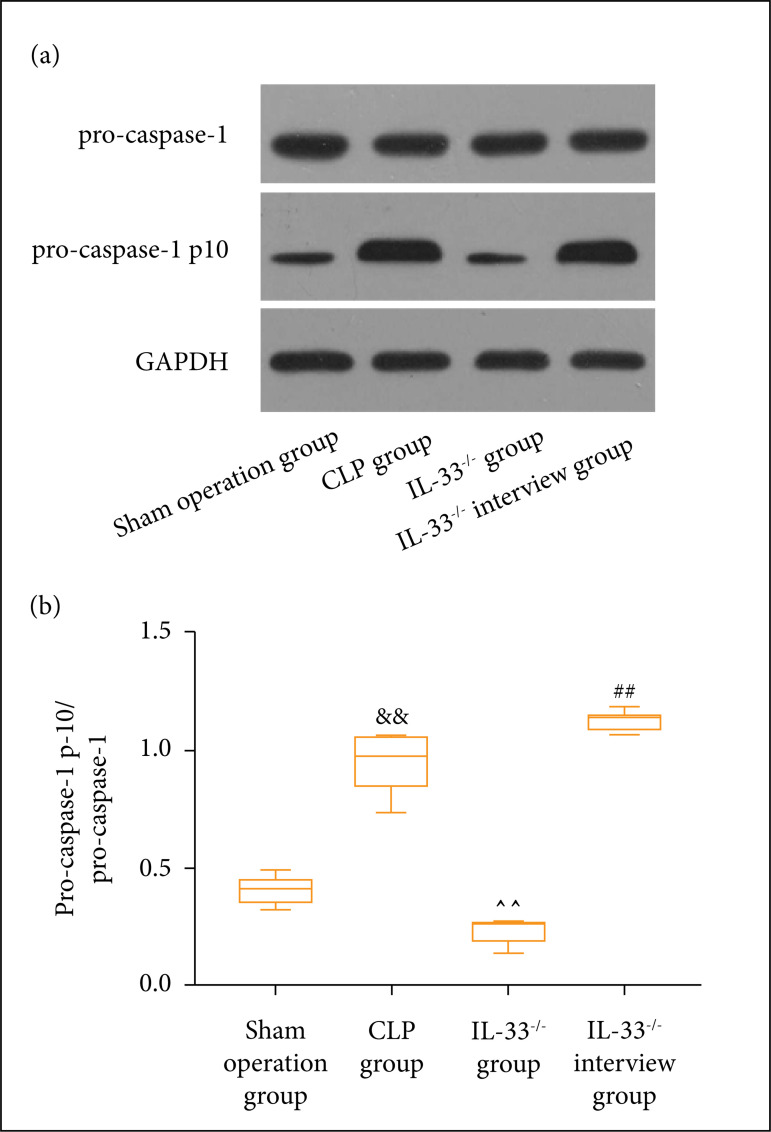
Expression level of pyroptosis-related proteins in macrophages of mice. **(a)** Bands; **(b)** statistical graph. The expression level of pro-caspase-1 p10 in macrophages of mice in sham operation, model, IL-33^-/-^,and IL-33^-/-^ intervention groups (n = 5); ^&&^p < 0.01 vs. sham operation group; ^^^^p < 0.01 *vs.* model group;^##^p < 0.01 vs. IL-33^-/-^ group.

Western blotting was employed to detect the expression level of pyroptosis-related proteins in macrophages in the peritoneal lavage fluid of the mice in each group. According to Fig. 5, the expression level of pro-caspase-1 p10 protein was clearly higher in the model group than the sham operation group (p < 0.01). In comparison to the IL-33^-/-^ group, IL-33^-/-^ intervention group exhibited a notably elevated expression level of pro-caspase-1 p10 protein (p < 0.01), whereas the IL-33^-/-^ group had a markedly lowered expression level of the same protein if compared to the model group (p < 0.01).

### Effect of IL-33 on NF-κB/p38 MAPK signaling pathway

**Figure 6:**
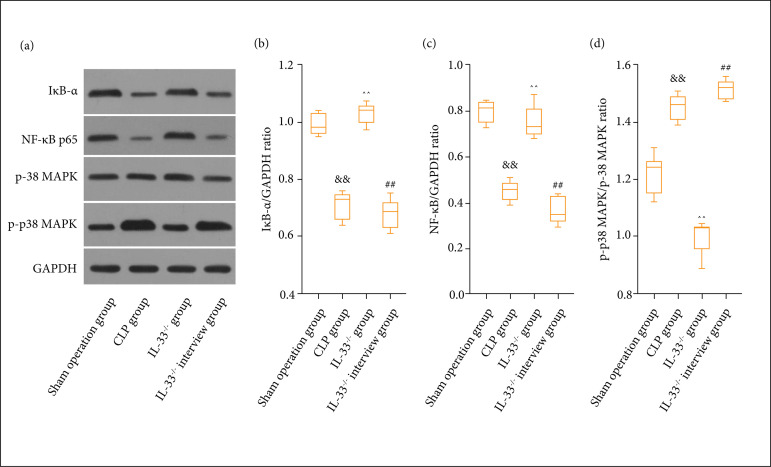
Expression level of NF-κB/p38 MAPK signaling pathway-related proteins in macrophages of mice determined by means of Western blotting. **(a)** Bands; **(b)** statistical graph of IκB-α; **(c)** statistical graph of NF-κB p65; (d) statistical graph of p-p38 MAPK. The expression levels of IκB-α and NF-κB p65 proteins of mice in sham operation, model, IL-33^-/-^, and IL-33^-/-^ intervention groups (n = 5); ^&&^p < 0.01 *vs.* sham operation group; ^^^^p < 0.01 *vs.* model group; ^##^p < 0.01 *vs.* IL-33^-/-^ group.

The expression levels of NF-κB/p38 MAPK signaling pathway-related proteins in the mice peritoneal lavage fluid were measured via Western blotting assay. It was found that, compared to the sham operation group, the expression levels of IκB-α, and NF-κB p65 proteins were clearly lowered (p < 0.01), while the expression level of p-p38 MAPK was overtly increased in the model group (p < 0.01). In comparison to IL-33^-/-^ group, IL-33^-/-^ intervention group exhibited a notably elevated expression level of p-p38 MAPK (p < 0.01) and visibly decreased expression levels of IκB-α and NF-κB p65 proteins (p < 0.01). The expression levels of IκB-α and NF-κB p65 proteins were clearly higher in the IL-33^-/-^ group than in the model group (p < 0.01), whereas the expression level of p-p38 MAPK was prominently lower in the IL-33^-/-^ group than in the model group (p < 0.01) (Fig. 6).

## Discussion

As a fatal organ dysfunction, caused by infection-induced host response dysregulation, sepsis remains the most common cause of intensive care unit (ICU) admission in the world, and accounts for almost 11% of all ICU cases in high-income countries, with a mortality rate of 18 to 35%^11^. Pyroptosis, a mode of cell death able to result in massive inflammatory responses, can release a large number of inflammatory factors, and trigger inflammatory cascade, which is a kind of caspase-1-dependent programmed cell death^12^. Hu *et al*.^13^ studied and discovered that the death from sepsis is closely associated with the severe inflammatory responses, and massive pyroptosis in the body. In this study, mouse models of sepsis were established *in vitro*, and the effects of IL-33 on the NF-κB/p38 MAPK signaling pathway and pyroptosis of macrophages in mice with sepsis were assessed. The results showed that: 1) IL-33 had a close relation to the occurrence and development of sepsis; 2) IL-33 could induce the pyroptosis of macrophages in mice with sepsis; and 3) IL-33 activated the NF-κB/p38 MAPK signaling pathway to up-regulate the expression of pro-caspase-1 p10, a pyroptosis-related protein in macrophages, thus mediating the pyroptosis of macrophages in mice with sepsis.

IL-33, a member of the IL-1 family, has gene sequence and structure similar to other members – IL-1β, and IL-18^14^. A study by Drake *et al*.^15^ denotes that full-length IL-33 is biologically active, but it will be converted into a highly active form under the action of protease, and IL-33 is inactivated after cleavage by caspase-1. In addition, IL-33 binds to ST2 to participate in immune responses. Nian *et al*.^16^ used ST2-knockout mice to construct the model of sepsis-induced injury and found that there are lymphocyte recruitment disorders in transgenic mice after modeling, without increases in the number of inflammatory factors and lymphocytes. In this study, IL-33^-/-^transgenic mice were prepared into models of sepsis, and it was found that, after modeling, the survival rate of IL-33^-/-^ transgenic mice was dramatically increased, while the content of inflammatory factors and the pyroptosis level in the body were obviously reduced. Research by Esquerdo *et al*.^17^ manifests that IL-33 is capableof promoting the lipopolysaccharide-induced release of inflammatory factors IL-1β, and IL-18 in macrophages, and the intraperitoneal injection of IL-33 increases the concentration of both Th2 cytokines and IL-5 and inflammatory factors IL-1β, and IL-18. Caspase-1 activity is also required for processing of cytokines like IL-18 and for the induction of the intracellular pathogen-induced cell death mechanism known as pyroptosis. Caspase-1 has autocatalytic activity during the induction phase of recruiting inflammasomes, leading to its fragmentation into p10 and p20 functional subunits. Inflammatory bodies mediate caspase-1 activation, leading to the production of IL-1β, and other inflammatory factors are important host responses that determine the disease. In our study, it was found that the expression of caspase-1 p10 in septic mice was significantly increased, and IL-33 knockout could significantly inhibit the formation of caspase-10 subunits in septic mice.

The occurrence and development of sepsis have close correlations with inflammatory responses mediated by the TLR signaling pathway. Activated TLR can activate the downstream kinases p38 MAPK and IκB and the transcription factor NF-κB^18^. P38 MAPK is a member of the MAPK family. The activated p38 MAPK signaling pathway can increase the level of p-p38 MAPK, thereby activating NF-κB, and mediating the occurrence and development of inflammatory responses in the body^19^,^20^. Previous studies have shown that IL-33 promotes radiculopathy by regulating the activation of MAPK and NF-κB, as well as the expression of inflammatory mediators in the spinal cord^21^. In addition, studies have shown that IL-33 can increase the expressions of IL-4 and IL-6 and also cause the activation of p38 MAPK and NF-κB in acute myeloid leukemia^22^. It suggests that IL-33 plays an important role in the inflammatory environment. The results of this study revealed that the NF-κB/p38 MAPK signaling pathway was activated in mice with sepsis. After modeling of sepsis in IL-33^-/-^ transgenic mice, the activation of the NF-κB/p38 MAPK signaling pathway was significantly inhibited; the pyroptosis of macrophages was suppressed; and the septic mice mortality rate was reduced. The aforementioned results strongly indicate that IL-33-induced pyroptosis of macrophages in mice with sepsis has a close correlation with the NF-κB/p38 MAPK signaling pathway. Using IL-33 as a therapeutic target and inhibiting the MAPK/NF-κB signaling pathway may become a possible way to treat severe inflammatory diseases, such as sepsis in the future. There are still some shortcomings in this study. The regulatory role of IL-33 in the first and second signals of inflammasomes during pyroptosis of macrophages in sepsis was not investigated deeply, which will be the direction and focus of subsequent studies.

## Conclusion

IL-33 facilitates the pyroptosis of macrophages in mice with sepsis, and increases the death rate of such mice by activating the NF-κB/p38 MAPK signaling pathway, providing a theoretical basis for the diagnosis and treatment of patients with sepsis in clinical practice.
